# Culture adaptation of malaria parasites selects for convergent loss-of-function mutants

**DOI:** 10.1038/srep41303

**Published:** 2017-01-24

**Authors:** Antoine Claessens, Muna Affara, Samuel A. Assefa, Dominic P. Kwiatkowski, David J. Conway

**Affiliations:** 1 London School of Hygiene and Tropical Medicine, London, UK; 2 Medical Research Council Unit The Gambia, Atlantic Road, Fajara, P.O. Box 273, Banjul, The Gambia; 3 The Wellcome Trust Sanger Institute, Hinxton, Cambridge, United Kingdom

## Abstract

Cultured human pathogens may differ significantly from source populations. To investigate the genetic basis of laboratory adaptation in malaria parasites, clinical Plasmodium falciparum isolates were sampled from patients and cultured *in vitro* for up to three months. Genome sequence analysis was performed on multiple culture time point samples from six monoclonal isolates, and single nucleotide polymorphism (SNP) variants emerging over time were detected. Out of a total of five positively selected SNPs, four represented nonsense mutations resulting in stop codons, three of these in a single ApiAP2 transcription factor gene, and one in SRPK1. To survey further for nonsense mutants associated with culture, genome sequences of eleven long-term laboratory-adapted parasite strains were examined, revealing four independently acquired nonsense mutations in two other ApiAP2 genes, and five in Epac. No mutants of these genes exist in a large database of parasite sequences from uncultured clinical samples. This implicates putative master regulator genes in which multiple independent stop codon mutations have convergently led to culture adaptation, affecting most laboratory lines of *P. falciparum*. Understanding the adaptive processes should guide development of experimental models, which could include targeted gene disruption to adapt fastidious malaria parasite species to culture.

All species adapt to their environment through the process of natural selection. In particular, many organisms grown in laboratory culture have adapted to an extent that they differ significantly from their original source populations. Given that many microbes occurring in nature are unable to be grown under culture conditions^1^,^2^, understanding the processes of culture adaptation may reveal key differences between cultivated and non-cultivated types. This knowledge should guide efforts to enable growth of fastidious organisms, by fine-tuning culture conditions or potentially by targeted genetic engineering to overcome metabolic barriers to growth. This is particularly important for enabling research on the biology of major human pathogens, including malaria parasites that are responsible for over half a million deaths each year^3^.

For over a century it has been possible to culture blood stages of different human malaria parasite species isolated from clinical infections for a few replication cycles in human erythrocytes *in vitro*^4^. Long-term continuous culture was first achieved for *Plasmodium falciparum* more than forty years ago^5^, but it has not been possible to continuously culture other human malaria parasite species, apart from two isolates of the zoonotic parasite *P. knowlesi* that have recently been adapted^6^,^7^. Moreover, only a few culture adapted *P. falciparum* lines have been used for most studies to date, and it is unclear how well they represent parasites in natural infections, as many clinical isolates do not show robust continuous growth *in vitro*^8^. Early studies of parasite chromosomal polymorphism among a few cultured parasite lines revealed deletion of a chromosomal subtelomeric region containing genes that are not required *in vitro*^9^. More recently, loss-of-function frameshift mutations in an *ApiAP2* transcription factor gene were detected in particular cultures that had lost ability to differentiate into sexual stages, enhancing asexual parasite replication^10^. Functional changes resulting from mutations in other genes may also be positively selected, and identification of these should reveal cellular and biochemical mechanisms that are important in the process of culture adaptation.

Here, genome sequence analyses identify malaria parasite genes in which mutations are associated with culture adaptation. Sequencing of multiple time point samples was conducted on new clinical isolates that adapted successfully to culture, and novel single nucleotide polymorphism (SNP) alleles were shown to have emerged, most of which lead to premature stop codons. Subsequently, a scan for nonsense mutation alleles in genome sequences of long-term adapted laboratory strains was conducted, and compared with sequences of a large global sample of uncultured clinical isolates. Together, these results have identified several putative master regulator genes in which multiple independent nonsense mutations are specifically associated with culture adaptation.

## Results

### Culture adaptation of new clinical isolates

**Figure 1:**
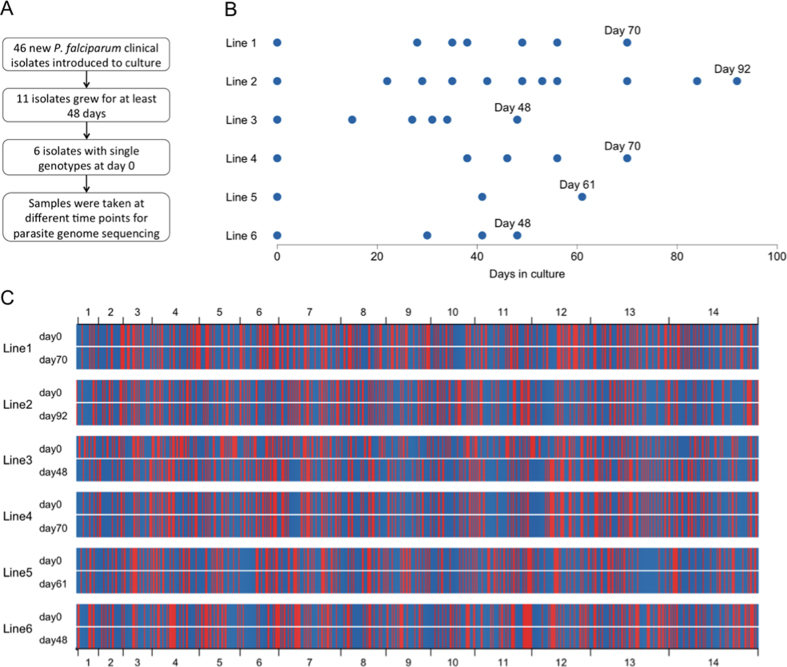
Culture of new *P. falciparum* clinical isolates and sampling for genome sequencing. (**A**) Flowchart showing numbers of isolates cultured and those selected for analysis. (**B**) Circles indicate culture time points of six single genotype isolates sampled for whole genome sequencing. (**C**) Genome-wide SNP haplotypes of these six isolates at the start and end of the culture periods. Each thin vertical line is a SNP (blue if it is identical to the 3D7 reference genome sequence, red if it is the alternative allele), shown in a concatenated linear scheme for all 14 chromosomes; for visual clarity, only one out of every 20 SNPs is shown. At the start of culture, the isolates were unrelated (each distinct from the others by between 5000 and 7000 detectable SNP differences). Five of the lines show unique genome-wide haplotypes that are unchanged throughout culture, but Line 3 was evidently contaminated by Line 4 (time course of replacement is shown in Fig. S1).

*P. falciparum* parasites from 46 different Gambian malaria patients were introduced into standard parasite culture conditions, out of which 11 (24%) of the isolates were successfully grown for at least 48 days. Of these, for analysis we selected six isolates that each contained single genotypes, as determined by PCR amplification and typing of the highly polymorphic *msp1* and *msp2* gene loci (Fig. 1A). Whole genome Illumina short-read sequencing was performed on a total of 35 time point samples from these six isolates (between three and 11 time points for each isolate, ranging from day 0 to day 92 of culture) (Fig. 1B and Table S1). Sequence read-pairs were mapped to the ~23 Mb *P. falciparum* 3D7 reference genome sequence, enabling detection of SNPs throughout coding regions of the core genome (94.7% of all genes). As an indication of sample quality, out of 944,270 of the MalariaGEN globally ascertained SNPs, at least 99.65% were successfully genotyped in each time point sample (www.malariagen.net/resource/20). At Day 0, each isolate had a distinct sequence that was unrelated to any other, and equally unrelated to the 3D7 reference genome sequence (Fig. 1C). This is expected, as *P. falciparum* genomes are normally diverse among different infections in West Africa, due to relatively high levels of transmission and recombination in the local parasite population^11^,^12^. By the end of the culture period, five isolates had retained their unique haplotypes, but line 3 had evidently been contaminated and overgrown by line 4 (Fig. 1C and Fig. S1) and so was removed from further analysis. In two of the isolates (lines 5 and 6), no emerging new sequence variant was detected at any point during the cultured periods, as any sequence read counts for alternative nucleotides remained at background levels that did not change significantly over time (Fig. S1).

**Figure 2:**
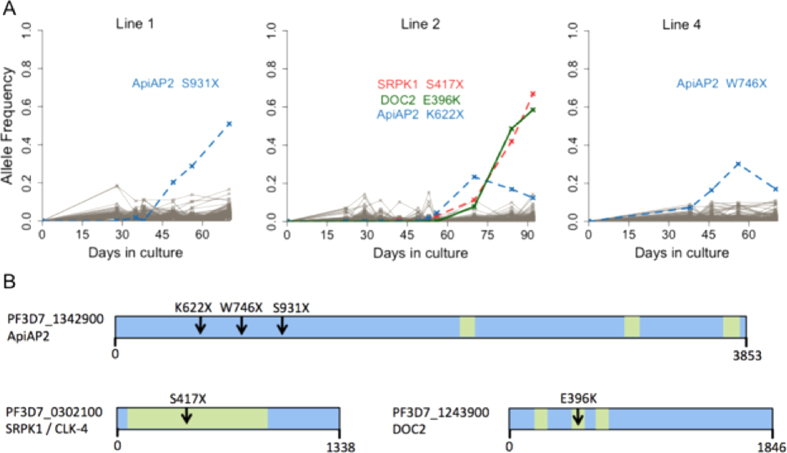
Selection of novel SNP alleles during culture adaptation of three unrelated *P. falciparum* clinical isolates in The Gambia. (**A**) Allele frequencies at each sampled time point were determined by alternative sequence read counts for each SNP, and novel alleles reaching a frequency of more than 20% over the time course are plotted in colour (dashed lines indicate nonsense mutant alleles). The temporal frequency changes for these five SNPs attained genome wide significance (P < 10^−9^ for each; read counts at each timepoint are given in Table S2). (**B**) Gene models with an arrow indicating the mutation position for each emerging SNP identified in panel A. Green boxes indicate predicted functional domains in each gene: AP2 domains in an *ApiAP2* transcription factor gene, the catalytic domain in the serine/threonine protein kinase gene *SRPK1*, and Calcium/lipid-binding C2 domains in *DOC2*. The numbers of codons in each gene are indicated underneath each scheme.

Three of the isolates (lines 1, 2 and 4) showed emergence of novel SNP alleles during culture, resulting from five independent mutations (Fig. 2A). The changes in frequencies of each of these were highly significant (P < 10^−9^ for each, remaining significant after Bonferroni correction for scanning all nucleotide positions in the genome; Table S2). In the first isolate (line 1), a single novel SNP allele emerged in an *ApiAP2* transcription factor gene (PF3D7_1342900) and increased in frequency so that the mutants were in a majority by the end of the culture period. This was a nonsense mutant leading to a stop codon S931X which would truncate the ApiAP2 protein by more than half its length (Fig. 2). In the second isolate (line 2), three novel SNP alleles emerged. The first was in the same *ApiAP2* transcription factor gene (PF3D7_1342900) that was noted in the line above, with a separate premature stop codon mutant K622X (Fig. 2). This mutant was then overtaken in frequency by parasites with two novel SNP alleles. These had a stop codon S417X in *SRPK1* (serine/threonine protein kinase gene, PF3D7_0302100) and a non-synonymous substitution E396K in *DOC2* (double C2-like domain-containing protein gene, PF3D7_1243900), likely to be within the same genome as they emerged in parallel and represented a majority by the end of the culture period (Fig. 2). In the third isolate (line 4), yet another independent stop codon mutant (W746X) of the same *ApiAP2* gene (PF3D7_1342900) emerged and increased in frequency until day 56 (Fig. 2).

Overall, four out of the five novel SNPs emerging during culture adaptation of these clinical isolates represented nonsense mutations. Of these, 3 occurred in different locations of the same *ApiAP2* transcription factor gene (under an expected Poisson distribution, P < 10^−10^). A Pfam search on the full gene sequence identified three predicted AP2 domains (codons 2362–2415, 3066–3120, and 3789–3841), all of which are downstream of the stop codons and will not be translated in the mutant parasites (Fig. 2B). Although the emergence of each of these novel SNPs during culture was highly statistically significant, none attained fixation during the culture periods, which covered a limited number of generations (*P. falciparum* asexual replicative cycle time of ~48 hours). Two of the ApiAP2 mutants stopped increasing in frequency later during culture (replaced by another mutant in line 2, but for unknown reasons in line 4), illustrating a need to examine parasites that have been culture adapted over longer periods to identify mutants that become fixed.

### Genes with loss-of-function mutations specific to culture adaptation

**Figure 3:**
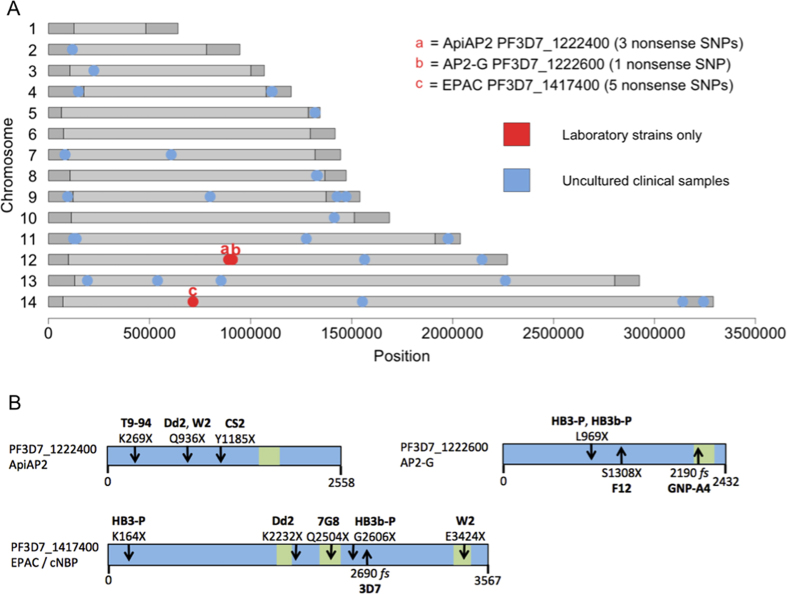
Nonsense mutations in long-term laboratory adapted *P. falciparum* strains are clustered in a few specific genes. (**A**) Genome-wide map with red dots indicating positions of genes having nonsense SNPs only in laboratory-adapted strains. These three genes (two *ApiAP2* genes and *Epac*) have nonsense SNP alleles detected among 11 laboratory strains (listed in Supplementary Tables S3 and S4) but not in sequences from uncultured clinical samples (blue dots indicate genes with nonsense SNPs in 1% or more of 2483 uncultured samples from the Pf3K project release 3.1 at www.malariagen.net/pf3k). The subtelomeric regions and internal non-core chromosomal regions (containing genes that are absent in most malaria parasite species) of the 3D7 reference genome are shaded in darker gray. (**B**) Models of the three genes with nonsense SNP alleles only in laboratory-adapted strains. Downward arrows show the positions of the premature stop codon alleles identified in each strain, while upward arrows indicate previously detected nonsense or frameshift (fs) mutations in two strains additional to those analysed here^10^ as well as the 3D7 strain reference genome sequence version 3.1 (http://www.genedb.org/Homepage/Pfalciparum). Green boxes indicate predicted catalytic and AP2 domains, and the numbers of codons in each gene are indicated underneath each scheme.

To examine whether nonsense mutations in particular genes are associated with historical culture adaptation, eleven genome sequences of long-term laboratory-adapted *P. falciparum* lines derived from seven original clinical isolates were studied (Fig. 3 and Table S3). These were compared with available sequence data from 2483 uncultured clinical samples (www.malariagen.net/pf3k pilot release 3) in which nonsense mutant alleles were very rare overall, and non-randomly associated with subtelomeric regions (Fig. 3A). Three genes contained nonsense mutant SNPs in the laboratory-adapted strains but never in uncultured clinical samples (Fig. 3A and Table S4). Two of these were *ApiAP2* genes, and one was *Epac* (annotated as a guanine nucleotide exchange factor) (Fig. 3B).

In one *ApiAP2* gene (PF3D7_1222400), three different nonsense mutation alleles were detected respectively in the long-term cultured lines T9-94, CS2 and the pair of related clones W2 and Dd2 (Fig. 3B). A novel nonsense mutation allele was also detected in the neighbouring *ApiAP2* gene (PF3D7_1222600) in clones of HB3 (Fig. 3B). This latter gene has been termed *AP2-G*, as it has a pivotal role in enabling differentiation to sexual stage gametocytes, and separate independent nonsense mutants have been described in sub-clones of the 3D7 parasite line^10^.

In the *Epac* gene, five unique nonsense mutation alleles were identified (Fig. 3B). Three of these are respectively at fixation in the long-term cultured lines Dd2, 7G8 and HB3-1a, whereas two others are respectively present in HB3-P and W2 but are not at fixation in these lines. In data from parasite clone tree culture experiments^13^, some subclones from HB3-P and W2 contained the premature stop codon allele while others did not. The nonsense mutation in *Epac* in the Dd2 clone has been previously noted^14^, as has the separate stop codon in W2^15^. In summary, most of the laboratory-adapted strains have evidently acquired one or more loss-of-function mutations in *Epac* or in an *ApiAP2* gene.

## Discussion

This study has identified key genomic changes associated with *P. falciparum* culture adaptation. First, new clinical isolates were cultured for up to three months, during which multiple samples were taken for whole-genome sequencing. Out of five novel SNPs clearly emerging during culture adaptation, four were a result of nonsense mutations. Three of these were in the same ApiAP2 transcription factor gene (*PF3D7_1342900*), a different mutation arising in each of three different cultured isolates. One isolate also had a nonsense mutation allele in a protein kinase gene (*SRPK1*). Following this, analysis of globally available data revealed three other genes in which nonsense mutation alleles are only seen in adapted, cloned, strains and not in clinical isolates. Two of these are other ApiAP2 genes, and one is the *Epac* gene encoding a guanine nucleotide exchange factor. Considering all results together, so it is remarkable that four particular genes (three members of the *ApiAP2* family and *Epac*) show multiple independent loss-of-function mutations in different cultured lines, and none in sequences of uncultured parasites.

Loss-of-function mutations have been shown to commonly occur during culture adaptation of bacteria^16^, and may be adaptive by removing an enzyme detrimental in the environment of interest, or by shutting off entire pathways. These beneficial mutations can act multiple steps away from the cellular function that they modulate, as noted for deletions in *E. coli* adapting to high temperatures^17^. By preventing unnecessary enzymatic reactions, the organism saves its resources for optimal growth in its current environment.

The four genes identified here with convergent nonsense mutants selected in culture probably have major roles in parasite growth. For the three repeatedly mutated *ApiAP2* transcription factor genes, it is likely that disruption would interfere with the expression of many other genes^18^, and may alter entire developmental pathways. Loss of function mutations in one of the *ApiAP2* genes (PF3D7_1222600, also termed *AP2-G*) in two laboratory strains were previously shown to prevent gametocytogenesis^10^,^19^. The independent nonsense mutation in this gene described here in another laboratory adapted line should have a similar effect, so that parasites with premature stop codons in this gene proportionally produce more asexual stages for faster growth in culture. The adjacent *ApiAP2* gene (PF3D7_1222400) with three independent nonsense mutations in laboratory strains identified here, in its intact state is predicted to encode a transcription factor with a very high number of gene targets^18^, and is the only *ApiAP2* in *P. falciparum* without a homologue detected in rodent malaria parasites^20^. The *ApiAP2* gene (PF3D7_1342900) with three different nonsense mutations identified in the new clinical isolates encodes a transcription factor preferentially interacting with G-boxes [(A/G)NGGGG(C/A)], which is a putative regulator of heat shock genes during the asexual blood stage cycle^18^,^21^ and is also expressed in other stages of the parasite life cycle^22^. Adaptation of asexual blood stage parasites to culture may involve improved tolerance of rapid temperature fluctuations occurring repeatedly during laboratory processing outside of culture incubators every few days.

In one of the cultured clinical isolates, parasites with a novel premature stop codon in *SRPK1* and with a nonsynonymous change in the *DOC2* gene outcompeted wild-type parasites and also began to replace the *ApiAP2* (PF3D7_1342900) mutant parasites. The gene product SRPK1 catalyzes phosphorylation of the parasite PfSR1 (Ser/Arg-rich) protein, involved in mRNA splicing in the nucleus^23^. The *SRPK1* gene transcript is expressed in *P. falciparum* asexual stages and at an even higher level in gametocytes^24^, and its orthologue is necessary for male gamete maturation in rodent malaria parasites^25^. Previous attempts to knock out the gene in a laboratory-adapted strain of *P. falciparum* by targeted disruption have not succeeded, suggesting that it was essential as appears the case for other related genes^26^. The growth shown by the *SRPK* mutant here indicates either that disruption is adaptive in certain conditions of initial culture establishment, or that it is tolerated in the context of a particular nonsynonymous alteration in *DOC2*, a gene that has been shown to affect microneme exocytosis which is important for erythrocyte invasion^27^.

Faster growth of asexual blood stage malaria parasites could be due to changes in various developmental parameters, including decreased cycle time, increased numbers of merozoites produced per cycle, or higher merozoite invasion rates into erythrocytes^28^. The complex process of merozoite invasion involves cAMP and Ca^2+^, which mediate signalling pathways regulating microneme secretion in the merozoite. An increase in cAMP apparently activates protein kinase A and Epac, the latter then activating Rap1 which increases cytosolic Ca^2+^ level through the phospholipase C pathway^29^. Nonsense mutations in the *Epac* gene have occurred in a large proportion of long-term adapted laboratory strains, and it is possible that its disrupted function is partly complemented by the role of protein kinase A in the culture environment. Epac may also have additional functions as a cyclic nucleotide binding protein^30^. Increased asexual growth can also result from a reduction in resources committed towards sexual stage gametocyte development, so a potential role in gametocytogenesis should also be considered for *Epac* and the *ApiAP2* genes identified here (apart from *AP2-G* for which this function is already known). To address this experimentally, assays need to be developed to reliably phenotype gametocytogenesis responses to relevant induction treatments in culture, as processes previously used have been crude and experimentally variable^31^.

Genomic analyses have revealed loss-of-function mutations in pathways that affect the replication of yeast under particular culture conditions^32^,^33^, including nutrient limitation^32^,^34^. Experiments with yeast have also shown that balancing selection may maintain subpopulations with and without a premature stop codon allele, growing in different parts of a culture flask^35^,^36^. It remains to be determined whether growth under different physical conditions, such as shaking rather than static incubation of culture flasks^37^, may influence the direction or intensity of selection on novel mutants of malaria parasites. It is notable that the *ApiAP2* gene with a different stop codon mutant emerging in each of three Gambian clinical isolates was intact in all long-term culture adapted lines, although some of these had premature stop codon alleles in either of two other *ApiAP2* genes. This suggests that not all alleles selected in the short term may go to fixation, and that other mutants might take over in a culture line over time, a pattern consistent with the trajectory of frequency changes seen in the clinical isolates here. A future priority is longer-term analysis of sequence evolution in culture, as this approach has given unique insights into adaptive processes in bacteria^38^,^39^, although numbers of generations that can be continuously followed in parasite culture are relatively limited due to the ~48 hour asexual cycle time. It is also possible that the particular functional loss selected in culture will depend on the parasite genomic background, which could be investigated by study of additional clinical isolates from different populations.

The present study focuses on single nucleotide variants that are robustly called, whereas development of methods to accurately detect novel indels and structural variants in Illumina sequences of *P. falciparum* cultures might have the potential to identify additional changes^40^. Although indels might cause frameshifts in other genes, it is nonetheless remarkable that, out of more than 5000 genes in the *P. falciparum* genome, only five (*Epac, SRPK1* and 3 *ApiAP2* genes) are identified to have nonsense SNP mutants causing premature stop codon alleles that have emerged in culture. With the exception of *SRPK1*, these genes all showed independent nonsense mutations in multiple strains. This suggests that the current study might have identified most of the genes that confer a very substantial growth advantage with a loss-of-function mutation. Although the long-term cultured laboratory strains were originally of diverse origins, almost all of them are now mutants having loss-of-function in *Epac* or *ApiAP2* genes. Understanding the impact of this profound convergent evolution in culture is vital, as the derived strains may have some limits as models of malaria parasites. It will be important to understand the impact of these adaptations, as this may guide the development of better experimental models for malaria, including culture adaptation of other malaria parasite species. Despite efforts undertaken in different laboratories over many years, long-term cultures of *P. vivax* have not yet been established, and culture of either *P. malariae* or *P. ovale* has been rarely attempted as it is regarded as unlikely to succeed. The results presented here encourage functional studies of parasites early during *ex vivo* cultivation, and raise the possibility that targeted deletion or editing of *Epac* or *ApiAP2* genes might provide a fast-track to culture adaptation of otherwise fastidious parasites.

## Materials and Methods

### Collection and processing of clinical isolates

The sample collection, laboratory culture and parasite genome sequencing for this study was reviewed and approved by the joint Ethics Committee of the Gambian Government and the MRC Gambia Unit. Written informed consent was obtained from all subjects or their attending guardian at the clinic before enrolment, with additional verbal assent of children being confirmed when guardians provided written consent. The methods were carried out in accordance with the relevant guidelines and regulations.

Patients with malaria were sampled from four health centers within the Greater Banjul area in The Gambia (The Royal Victoria Teaching Hospital in Banjul, Brikama Health Centre, the Jammeh Foundation for Peace Hospital in Serekunda, and the MRC outpatients clinic in Fajara), between September 2009 and November 2010. Each patient had axillary temperature of >37.5 °C or a history of fever in the previous 48 h, and *P. falciparum* parasitaemia of >5000 per microliter as estimated by thick blood film examination, with a thin blood film examination to confirm the parasite species as *P. falciparum* alone. A heparinized venous blood sample of up to 5 ml was collected from each patient and centrifuged to remove the plasma. Separation of the erythrocytes from leukocytes was performed using Nycoprep density gradient centrifugation, following which erythrocytes were washed and resuspended at 50% hematocrit in RPMI 1640 growth medium, supplemented with 25 mmol/L HEPES, 2 mmol/L l-glutamine, 25 mmol/L D-glucose, 25 mg gentamicin/L, 10 mg hypoxanthine/L and 10% albumax. Infected erythrocytes were either put directly into *in vitro* culture or cryopreserved for culture at a later date.

### Parasite culture adaptation and sampling for genome sequencing

Clinical isolates were cultured in complete RPMI 1640, using standard methods^5^, with slight modifications as follows. Isolates were incubated at 37 °C in a modular incubator chamber containing a gas mixture of 1% O_2_, 3% CO_2_ and 96% N_2_. Samples were checked every day by thin blood film examination, and fresh medium and uninfected blood group O erythrocytes were added when necessary to maintain hematocrit at 2% and parasitemia below 10% of erythrocytes infected. Isolates were grown in culture for up to 92 days, with samples for parasite genome sequence analysis being taken at multiple time points during the culture period, depending on the parasitemia and volume of culture material available at various times. DNA was extracted from 200 μl packed erythrocytes using the QIAamp DNA Blood Mini Kit (Qiagen). To identify isolates that were apparently single genotype infections, the pre-culture (day 0) DNA sample from each isolate was screened by genotyping highly polymorphic repeat regions of *msp1* and *msp2* genes^41^.

### Genomic DNA library preparation and whole-genome sequencing

Library preparation, sequencing and quality control was completed following protocols as described previously^11^. Single Nucleotide Polymorphisms (SNPs) were called by a pipeline of the MalariaGEN consortium (version 4.0, www.malariagen.net/data/pf3k-pilot-data-release-4) following methods based on those previously published^11^. This process involved a global sample of *P. falciparum* genomes, including the 35 cultured timepoints sampled here, being mapped to the 3D7 reference genome sequence version 3.1 (http://www.genedb.org/Homepage/Pfalciparum) with the BWA algorithm. Samtools mpileup called a total of 4,305,966 potential SNPs (positions that appear to contain a variation from the reference allele). Low quality SNPs were filtered out if any of the following condition was not met, for each position: base quality score threshold superior to 27, inside coding regions, represented by more than 10 reads in a single sample or more than 1% of all reads, total coverage is within the 5^th^ and 99.5^th^ percentiles of the coverage distribution, uniqueness score greater than 26 (further detailed in the MalariaGEN process report www.malariagen.net/data). A final global dataset of 944,270 high-quality SNPs was used (https://www.malariagen.net/resource/20), covering coding regions of the core genome, with highly polymorphic gene families *var, rif* and *stevor* being excluded from analysis.

### Data analysis

Novel SNPs arising during culture adaptation were identified at nucleotide positions where there was no minor allele detected at day 0, but where an alternative nucleotide appeared in an isolate during culture and subsequently increased in frequency to represent more than 20% of the mapped sequence reads. The quality of the mapped sequence reads for each of these cases were inspected visually using the LookSeq software^42^. To compare with the sequences in long-term laboratory adapted strains, a global dataset of clinical *P. falciparum* samples was examined, using the Pf3K project release 3.1 (www.malariagen.net/projects/parasite/pf3k). This contained sequences of 2483 *P. falciparum* clinical samples of diverse geographical origin, after removing 29 that were annotated as “culture adapted”, with the same pipeline for SNP calling used as above (MalariaGEN v 4.0). Different types of SNP types in coding regions (synonymous, non-synonymous, nonsense) were determined with the R package VariantAnnotation^43^. All statistical analyses and graphs were generated using R (http://r-project.org).

## Additional Information

**How to cite this article**: Claessens, A. *et al*. Culture adaptation of malaria parasites selects for convergent loss-of-function mutants. *Sci. Rep.* **7**, 41303; doi: 10.1038/srep41303 (2017).

**Publisher's note:** Springer Nature remains neutral with regard to jurisdictional claims in published maps and institutional affiliations.

## Supplementary Material

Supplementary Tables and Figure
