# Supplementary material for: Culture adaptation of malaria parasites selects for convergent loss-of-function mutants
Source: Sci Rep. 2017 Jan 24;7:41303. doi: 10.1038/srep41303 (PMC5259787; doi:10.1038/srep41303)
Supplement: Supplementary Tables and Figure [file srep41303-s1.doc]

**Culture adaptation of malaria parasites selects for convergent loss-of-function mutants**

Antoine Claessens^a,b,1,*^, Muna Affara^b,1^, Samuel A Assefa^a^, Dominic P Kwiatkowski^c^ and David J Conway^a,*^

^a^ London School of Hygiene and Tropical Medicine, London, UK; ^b^Medical Research Council Unit, Fajara, Banjul, The Gambia; ^c^ The Wellcome Trust Sanger Institute, Hinxton, Cambridge, United Kingdom.

^1^ These authors contributed equally to this work.

^*^Corresponding authors: david.conway@lshtm.ac.uk and [antoineclaessens@gmail.com](mailto:antoineclaessens@gmail.com)

**Table S1.** Illumina short read sequences from multiple timepoints of six Gambian *Plasmodium falciparum* clinical isolates grown in culture for up to three months. Accession numbers and coverage of mapping to the 3D7 reference genome are shown. All genome-wide SNP genotype calls together with guidelines for data use are given in a dedicated MalariaGEN project page https://www.malariagen.net/resource/20 (to be released prior to publication)

| **Line Number** | **Days in culture** | **MalariaGEN code** | **ENA** | **Total reads** | **Mapped reads** | **Coverage (fold)** |
| --- | --- | --- | --- | --- | --- | --- |
| **Line 1** | 0 | PA0122-C | ERS036646 | 5373012 | 4475175 | 14.8 |
| Line 1 | 28 | PA0123-C | ERS036647 | 23012742 | 21939514 | 72.5 |
| Line 1 | 35 | PA0124-C | ERS036648 | 23561682 | 22465232 | 74.2 |
| Line 1 | 38 | PA0125-C | ERS036649 | 20710460 | 19752521 | 65.3 |
| Line 1 | 49 | PA0126-C | ERS036650 | 18796496 | 17903231 | 59.2 |
| Line 1 | 56 | PA0127-C | ERS036651 | 31992830 | 30482853 | 100.7 |
| Line 1 | 70 | PA0128-CW | ERS074126 | 17886528 | 17056701 | 56.4 |
| **Line 2** | 0 | PA0108-C | ERS036625 | 5349042 | 4770755 | 15.8 |
| Line 2 | 22 | PA0109-C | ERS036626 | 41923480 | 41295644 | 136.5 |
| Line 2 | 29 | PA0110-C | ERS036627 | 25474770 | 25111054 | 83.0 |
| Line 2 | 35 | PA0111-C | ERS036628 | 25585634 | 25585634 | 84.5 |
| Line 2 | 42 | PA0112-C | ERS036629 | 15688408 | 15453610 | 51.1 |
| Line 2 | 49 | PA0113-C | ERS036630 | 35562878 | 34996658 | 115.6 |
| Line 2 | 53 | PA0114-C | ERS157490 | 22378305 | 21637583 | 71.5 |
| Line 2 | 56 | PA0115-C | ERS036631 | 17471278 | 17204633 | 56.9 |
| Line 2 | 70 | PA0116-C | ERS036632 | 35106858 | 34579784 | 114.3 |
| Line 2 | 84 | PA0274-C | ERS074123 | 22310954 | 21977901 | 72.6 |
| Line 2 | 92 | PA0275-C | ERS074124 | 19902788 | 19603297 | 64.8 |
| **Line 3** | 0 | PA0129-C | ERS036653 | 25301532 | 23614639 | 78.0 |
| Line 3 | 15 | PA0130-C | ERS036654 | 22852168 | 21727816 | 71.8 |
| Line 3 | 27 | PA0131-C | ERS036656 | 20619316 | 19595655 | 64.8 |
| Line 3 | 31 | PA0132-C | ERS036657 | 30117196 | 28620930 | 94.6 |
| Line 3 | 34 | PA0133-CW | ERS074127 | 24020532 | 22869692 | 75.6 |
| Line 3 | 48 | PA0276-C | ERS074128 | 19018806 | 18748635 | 62.0 |
| **Line 4** | 0 | PA0117-C | ERS036633 | 2400640 | 1884289 | 6.2 |
| Line 4 | 38 | PA0118-C | ERS157482 | 22096733 | 21334137 | 70.5 |
| Line 4 | 46 | PA0119-C | ERS036634 | 44014412 | 43376864 | 143.3 |
| Line 4 | 56 | PA0120-C | ERS036635 | 29369732 | 28923836 | 95.6 |
| Line 4 | 70 | PA0121-C | ERS036636 | 31477398 | 31027772 | 102.5 |
| **Line 5** | 0 | PA0107-C | ERS016702 | 10522868 | 8618429 | 28.5 |
| Line 5 | 41 | PA0107-CW | ERS016703 | 60823912 | 59112125 | 195.3 |
| Line 5 | 61 | PA0107-CW2 | ERS016704 | 52694194 | 51454205 | 170.0 |
| **Line 6** | 0 | PA0106-C | ERS016698 | 38955976 | 36555036 | 120.8 |
| Line 6 | 30 | PA0106-CW | ERS016699 | 60405774 | 58508909 | 193.3 |
| Line 6 | 41 | PA0106-CW2 | ERS016700 | 42363784 | 41165796 | 136.0 |
| Line 6 | 48 | PA0106-CW3 | ERS016701 | 53366854 | 51858753 | 171.4 |

Note: Blood samples for lines 1 to 6 were collected on 8 Nov 2010, 4 Oct 2010, 30 Aug 2010, 30 Aug 2010, 10 Sep 2009, 23 Oct 2009, respectively.

**Table S2.** Sequence read counts of novel SNP alleles emerging during culture of *P. falciparum* clinical isolates. A colour scheme is added for visual clarity which highlights increasing allele frequency (from red to green).

| **Chromosome** | | | Pf3D7_13_v3 | Pf3D7_13_v3 | Pf3D7_13_v3 | Pf3D7_03_v3 | Pf3D7_12_v3 |
| --- | --- | --- | --- | --- | --- | --- | --- |
| **Position** | | | 1692106 | 1691733 | 1691178 | 115319 | 1839374 |
| **REF** | | | T | C | G | C | C |
| **ALT** | | | A | T | C | A | T |
| **gene ID** | | | PF3D7_1342900 | PF3D7_1342900 | PF3D7_1342900 | PF3D7_0302100 | PF3D7_1243900 |
| **Annotation** | | | transcription factor with AP2 domain(s) (ApiAP2) | transcription factor with AP2 domain(s) (ApiAP2) | transcription factor with AP2 domain(s) (ApiAP2) | Serine/threonine protein kinase (SRPK1 / CLK-4) | double C2-like domain-containing protein (DOC2) |
| **Amino acid** | | | K622X | W746X | S931X | S417X | E396K |
| **Line 1** | **Day 0** | Ref | 33 | 25 | **25** | 35 | 28 |
|  |  | Alt | 0 | 0 | **0** | 1 | 0 |
|  | **Day 28** | Ref | 78 | 58 | **62** | 62 | 37 |
|  |  | Alt | 0 | 0 | **0** | 0 | 0 |
|  | **Day 35** | Ref | 60 | 54 | **58** | 60 | 48 |
|  |  | Alt | 0 | 0 | **1** | 0 | 0 |
|  | **Day 38** | Ref | 62 | 47 | **53** | 59 | 45 |
|  |  | Alt | 0 | 0 | **0** | 0 | 0 |
|  | **Day 49** | Ref | 49 | 50 | **47** | 51 | 37 |
|  |  | Alt | 0 | 0 | **12** | 0 | 0 |
|  | **Day 56** | Ref | 97 | 103 | **62** | 92 | 76 |
|  |  | Alt | 0 | 0 | **25** | 0 | 0 |
|  | **Day 70** | Ref | 99 | 77 | **26** | 81 | 57 |
|  |  | Alt | 0 | 0 | **39** | 0 | 0 |
| **Line2** | **Day 0** | Ref | **97** | 90 | 88 | 81 | **54** |
|  |  | Alt | **0** | 0 | 0 | **0** | **0** |
|  | **Day 22** | Ref | **155** | 136 | 140 | **121** | **56** |
|  |  | Alt | **0** | 0 | 0 | **0** | **0** |
|  | **Day 29** | Ref | **68** | 78 | 75 | **79** | **39** |
|  |  | Alt | **0** | 0 | 0 | **0** | **0** |
|  | **Day 35** | Ref | **136** | 113 | 98 | **116** | **81** |
|  |  | Alt | **0** | 0 | 0 | **0** | **0** |
|  | **Day 42** | Ref | **294** | 281 | 296 | **278** | **195** |
|  |  | Alt | **0** | 0 | 0 | **2** | **0** |
|  | **Day 49** | Ref | **123** | 115 | 100 | **102** | **79** |
|  |  | Alt | **0** | 0 | 0 | **0** | **0** |
|  | **Day 53** | Ref | **292** | 294 | 243 | **272** | **184** |
|  |  | Alt | **5** | 0 | 0 | **2** | **0** |
|  | **Day 56** | Ref | **260** | 286 | 252 | **292** | **178** |
|  |  | Alt | **12** | 0 | 0 | **9** | **0** |
|  | **Day 70** | Ref | **82** | 124 | 101 | **95** | **71** |
|  |  | Alt | **25** | 0 | 0 | **12** | **6** |
|  | **Day 84** | Ref | **113** | 119 | 105 | **76** | **38** |
|  |  | Alt | **23** | 0 | 0 | **55** | **36** |
|  | **Day 92** | Ref | **106** | 93 | 94 | **40** | **22** |
|  |  | Alt | **15** | 0 | 0 | **81** | **31** |
| **Line 4** | **Day 0** | Ref | 99 | **77** | 68 | 88 | 45 |
|  |  | Alt | 0 | **0** | 0 | 0 | 0 |
|  | **Day 38** | Ref | 164 | **155** | 158 | 158 | 121 |
|  |  | Alt | 0 | **12** | 0 | 0 | 0 |
|  | **Day 46** | Ref | 132 | **127** | 147 | 126 | 79 |
|  |  | Alt | 0 | **25** | 0 | 0 | 0 |
|  | **Day 56** | Ref | 93 | **67** | 116 | 74 | 47 |
|  |  | Alt | 0 | **29** | 0 | 0 | 0 |
|  | **Day 70** | Ref | 93 | **78** | 76 | 79 | 51 |
|  |  | Alt | 0 | **16** | 0 | 0 | 0 |
| **Line 5** | **Day 0** | Ref | 46 | 37 | 39 | 40 | 15 |
|  |  | Alt | 0 | 0 | 0 | 0 | 0 |
|  | **Day 41** | Ref | 306 | 247 | 220 | 261 | 86 |
|  |  | Alt | 0 | 0 | 0 | 0 | 0 |
|  | **Day 61** | Ref | 231 | 235 | 180 | 173 | 82 |
|  |  | Alt | 0 | 0 | 0 | 0 | 0 |
| **Line 6** | **Day 0** | Ref | 163 | 117 | 106 | 118 | 44 |
|  |  | Alt | 0 | 0 | 0 | 0 | 0 |
|  | **Day 30** | Ref | 322 | 294 | 187 | 275 | 106 |
|  |  | Alt | 0 | 0 | 0 | 0 | 0 |
|  | **Day 41** | Ref | 180 | 138 | 116 | 131 | 44 |
|  |  | Alt | 0 | 0 | 0 | 0 | 0 |
|  | **Day 48** | Ref | 284 | 290 | 217 | 249 | 71 |
|  |  | Alt | 0 | 1 | 0 | 0 | 0 |

**Table S3.** Origins of long-term cultured laboratory strains of *Plasmodium falciparum* from which genome sequences were analysed.

| **Clone** | **Country of origin** | **Additional information and references** |
| --- | --- | --- |
| **3D7** | Africa | 1 |
| **IT** | Brazil | 2 |
| **CS2** |  | Clone of E8B, which itself is a clone of IT. ^3^ |
| **T994** | Thailand | 4 |
| **7G8** | Brazil | Clone used for the cross 7G8xGB4 ^5^ |
| **GB4** | Ghana | Clone used for the cross 7G8xGB4 ^5^ |
| **W2** | Indochina | 6 |
| **Dd2** | Indochina | Clone derived from W2, and used for the cross HB3xDd2 ^7^ |
| **HB3-cross** | Honduras | Clone used in HB3x3D7 and HB3xDd2 crosses ^7^ |
| **HB3b-P** | Honduras | HB3 cultured for an unknown period of time at Sanger. It was used as parent for clone tree experiment ^8^ |
| **HB3-P** | Honduras | Clone derived from HB3b-P. ^8^ |

References

1. Walliker, D. *et al.* Genetic analysis of the human malaria parasite Plasmodium falciparum. *Science* **236,** 1661–6 (1987).

2. Udeinya, I. J., Graves, P. M., Carter, R., Aikawa, M. & Miller, L. H. Plasmodium falciparum: effect of time in continuous culture on binding to human endothelial cells and amelanotic melanoma cells. *Exp. Parasitol.* **56,** 207–14 (1983).

3. Rogerson, S. J. Chondroitin sulfate A is a cell surface receptor for Plasmodium falciparum-infected erythrocytes. *J. Exp. Med.* **182,** 15–20 (1995).

4. Thaithong, S. *et al.* Pyrimethamine resistant mutations in Plasmodium falciparum. *Mol. Biochem. Parasitol.* **52,** 149–57 (1992).

5. Hayton, K. & Su, X.-Z. Drug resistance and genetic mapping in Plasmodium falciparum. *Curr. Genet.* **54,** 223–39 (2008).

6. Oduola, A. M. *et al.* Plasmodium falciparum: cloning by single-erythrocyte micromanipulation and heterogeneity in vitro. *Exp. Parasitol.* **66,** 86–95 (1988).

7. Wellems, T. E. *et al.* Chloroquine resistance not linked to mdr-like genes in a Plasmodium falciparum cross. *Nature* **345,** 253–5 (1990).

8. Claessens, A. *et al.* Generation of Antigenic Diversity in Plasmodium falciparum by Structured Rearrangement of Var Genes During Mitosis. *PLoS Genet.* **10,** (2014).

**Table S4.** Nonsense mutation alleles identified genome sequences of long-term *P. falciparum* laboratory adapted lines (strains listed in Table S3).

| **Chrom** | **Coordinate** | **Ref** | **Alt** | **Mutation** | **Strain** | **Gene ID** | **Annotation** | **Stop codon frequency in C.I.** |
| --- | --- | --- | --- | --- | --- | --- | --- | --- |
| 14 | 715197 | A | T | *K164X* | HB3-P | PF3D7_1417400 | Epac (cyclic Nucleotide Binding Protein) | 0.000 |
| 14 | 721986 | A | T | K2232X | Dd2 |
| 14 | 722802 | C | T | Q2504X | 7G8 |
| 14 | 723108 | G | T | G2606X | HB3-1a |
| 14 | 725564 | G | T | *E3424X* | W2 |
| 12 | 910108 | T | A | L969X | HB3-P, HB3b-P | PF3D7_1222600 | AP2-G | 0.000 |
| 12 | 891385 | A | T | K269X | T9-94 | PF3D7_1222400 | ApiAP2 family | 0.000 |
| 12 | 893386 | C | T | Q936X | Dd2, W2 |
| 12 | 894135 | C | A | Y1185X | CS2 |
| 13 | 2550469 | G | T | E835X* | IT, Dd2, W2, CS2, T994 | PF3D7_1363500 | DNase I-like protein, putative | 0.000 |
| 10 | 1442291 | C | T | Q235X | 7G8 | PF3D7_1036500 | probable protein, unknown function | 0.002 |
| 2 | 834445 | G | A | W172X | Dd2 | PF3D7_0220700 | Plasmodium exported protein (hyp9), unknown function | 0.02 |
| 3 | 70379 | A | T | L514X | IT, Dd2, W2, CS2, T994 | PF3D7_0301000 | acyl-CoA synthetase (ACS2) | 0.02 |
| 11 | 1978248 | A | T | R573X | IT, CS2, T9-94 | PF3D7_1149200 | ring-infected erythrocyte surface antigen | 0.118 |
| 13 | 853949 | A | T | K1430X | Dd2, W2 | PF3D7_1320700 | conserved Plasmodium protein, unknown function | 0.253 |
| 9 | 1427653 | G | A | Q92X* | IT, HB3, Dd2, W2, CS2, T9-94 | PF3D7_0936000 | ring-exported protein (REX2) | 0.654 |
| 9 | 1427697 | G | T | S77X | ALL HB3 |

Mutants in italics are present as a mixture, all others are fixed in the strain indicated. Nonsense mutations located only 1 or 2 amino acids before the canonical stop codon are indicated by asterisks. Note that the 3D7 reference strain shows a frameshift mutation in the *Epac* gene.

**Figure S1**

**
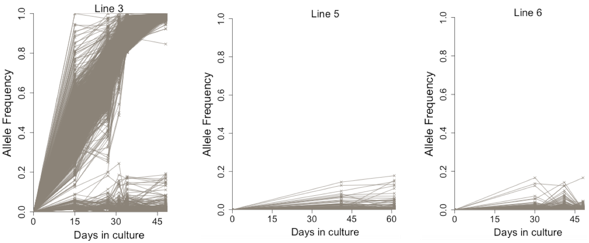
**

Supplementary Figure 1. Allele frequencies for line 3, line 5 and line 6. The progressive replacement of line 3 alleles by line 4 alleles is evident (as shown in Fig 1C), indicating that line 4 is a faster grower than line 3. In line 5 and 6, no allele significantly increased in frequency during the culture adaptation period.
